# LncRNA Snhg1, a non-degradable sponge for miR-338, promotes expression of proto-oncogene CST3 in primary esophageal cancer cells

**DOI:** 10.18632/oncotarget.16189

**Published:** 2017-03-14

**Authors:** Yan Yan, Qingxia Fan, Liping Wang, Yue Zhou, Jianhua Li, Kun Zhou

**Affiliations:** ^1^ Department of Oncology, The First Affiliated Hospital of Zhengzhou University, Zhengzhou, China; ^2^ Department of Medical Imaging, The First Affiliated Hospital of Zhengzhou University, Zhengzhou, China; ^3^ Department of General Surgery, The First Affiliated Hospital of Zhengzhou University, Zhengzhou, China; ^4^ Department of Thoracic Surgery, The First Affiliated Hospital of Zhengzhou University, Zhengzhou, China

**Keywords:** esophageal cancer, competing endogenous RNA, lncRNA Snhg1, miR-338, cystatin C

## Abstract

Competing endogenous RNA (ceRNA) is a newly proposed mechanism that describes a crosstalk among lncRNAs, mRNAs and their shared miRNAs. In this study, the role of miR-338-3p (miR-338) in the progression of esophageal cancer and its involve in the ceRNA regulatory circuit lncRNA-Snhg1/CST3 were explored. MiR-338 displayed a 30% decreased expression in esophageal squamous cell carcinoma tissues compared with the adjacent. Then, proto-oncogene CST3 was predicted and validated as a target gene of miR-338. Gain-and-loss-function experiments indicated that miR-338 suppressed expression of CST3 protein (also Cystatin C, CysC), promoted expression of apoptotic proteins caspase-8/3, attenuated esophageal carcinoma cell growth and induced its apoptosis. In addition, lncRNA-Snhg1 was significantly upregulated in esophageal carcinoma tissues and promoted esophageal carcinoma cell growth. Furthermore, our results from bioinformatics, luciferase reporter gene and RNA pull-down assays indicated that Snhg1 could be directly bound by miR-338. Snhg1 acted as a non-degradable sponge to relieve the suppression on CST3 caused by miR-338. In conclusion, lncRNA-Snhg1 promoted cell proliferation by acting as a non-degradable sponge for the tumor suppressor miR-338 in esophageal cancer cells.

## INTRODUCTION

Cystatins are tight-binding inhibitors against C1 cysteine proteases that regulate multiple physiological and pathological process [1]. All the members of Cystatins superfamily are categorized into three groups, including intracellular Cystatins A and B (type 1), secreted Cystatins C, D, E/M, F, G, H, S, SA, and SN (type 2), and kininogens (type 3) [1–3]. CysC, encoded by CST3 gene in human, is the most abundant type 2 cystatin. CysC was showed to target and suppress Cathepsins (especially Cathepsin and D) and promote caspases mediated cell death [4–6]. CysC was reported as a prognostic marker in several forms of cancer. For example, high serum CysC level was to closely linked to poor prognosis in colorectal cancer patients and melanoma metastasis [7, 8]. Lower CysC expression levels implied higher degree of prostate cancer and glioma, and were associated with poor prognosis of breast cancer [9, 10]. The exact role of CysC and its regulatory network in the progression of human esophageal cancer remain largely undetermined.

Non-coding RNAs (ncRNAs) are functionally important RNA transcripts that are not translated into proteins. NcRNAs include highly abundant RNAs such as transfer RNAs (tRNAs) and ribosomal RNAs (rRNAs), as well as relatively new found classes including small RNAs, such as microRNAs (miRNAs), and the long ncRNAs (lncRNAs) and circular RNAs (circRNAs). MiRNAs negatively regulate expression of their target genes at the post-transcriptional level [11]. LncRNAs precisely control gene expression at a variety of levels, such as transcriptional repression, alternative splicing, stability mediation, and translation regulation [12]. Interactions between ncRNAs, as well as with mRNAs, are recently pushed to the most forefront [13]. Competing endogenous RNA (ceRNA) is a currently proposed mechanism that describes a crosstalk among specific miRNAs, mRNAs and lncRNAs/circRNAs. In this mechanism, lncRNAs/circRNAs compete for the same pool of miRNA with specific mRNAs, thereby de-suppressing the mRNAs [14, 15]. There have been some validated ceRNA regulators that are invovled in several important physiological and pathological processes. For example, linc-MD1, a myogenic lncRNA, promotes muscle growth and differentiation by positively regulating expression of mastermind-like protein 1 (MAML1) and myocyte-specific enhancer factor 2C (MEF2C) via sponging miR-133 and miR-135 [16].

MiR-338-3p (also miR-338) was a proven suppressor in several cancers, including hepatocellular carcinoma, neuroblastoma and gastric cancer [17–19]. There is a lack of information about the exact role of miR-338 in the progression of esophageal cancer. In this study, we explored the effect of miR-338 on the processes of cell proliferation and apoptosis and clarified its interaction with lncRNA-Snhg1 in primary esophageal squamous cell carcinoma.

## RESULTS

### MiR-338 directly targeted CST3 and increased the apoptosis of human primary esophageal cancer cells

**Figure 1:**
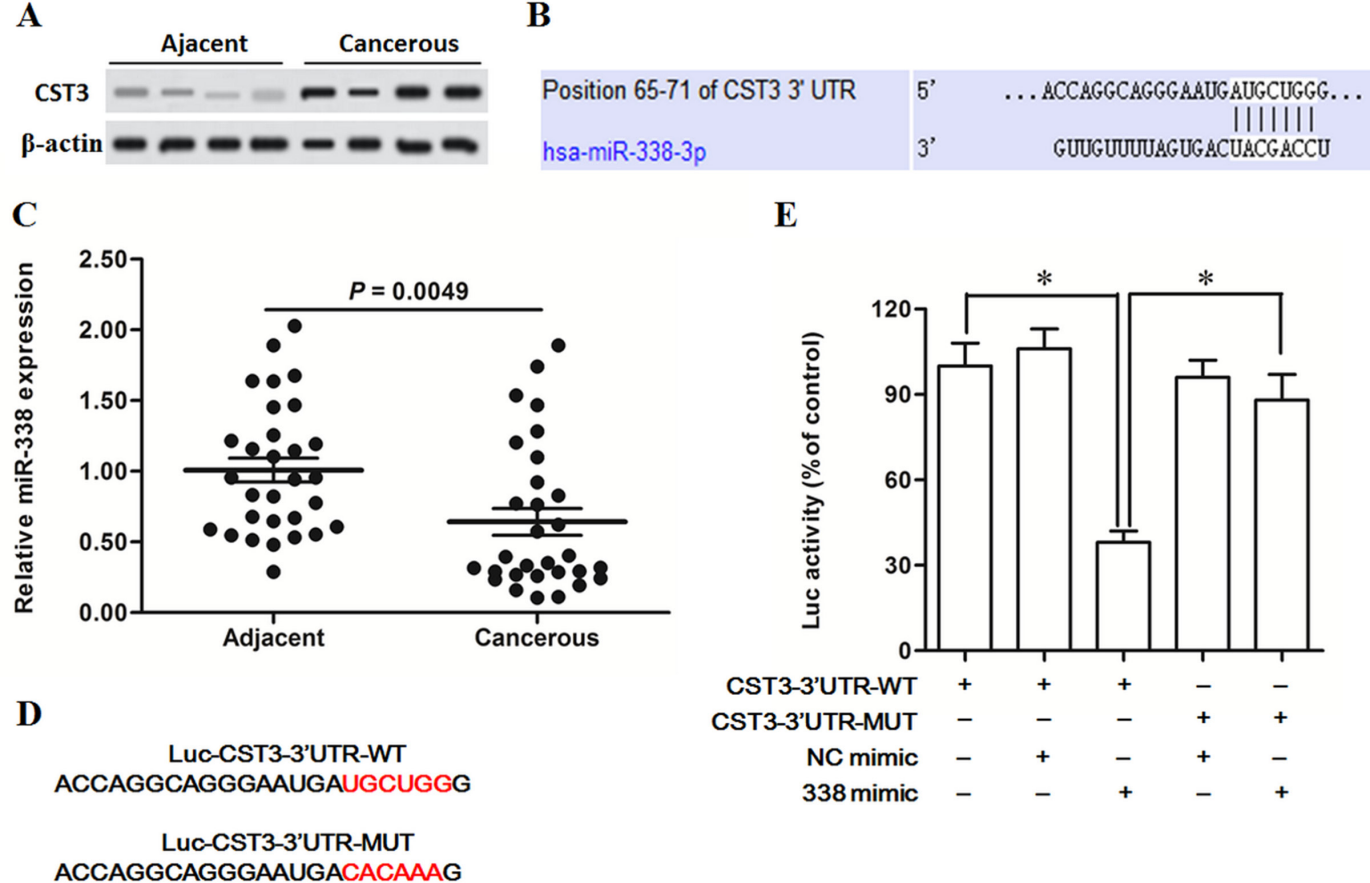
MiR-338 participates in the post-transcriptional suppression of CST3 gene (**A**) CST3 protein was upregulated in the esophageal squamous cell carcinoma tissue (*n* = 4). (**B**) TargetScan output revealed that miR-338 bound with CST3 mRNA sequence at the 3′ UTR. (**C**) MiR-338 was downregulated in the esophageal carcinoma tissue. Esophageal carcinoma tissues and matched adjacent tissues were isolated from 30 patients with primary esophageal squamous cell carcinoma. MiR-338 expression was detected with qPCR. (**D**) The sequences of wild type (WT) and mutated (MUT) CST3 mRNA 3′ UTR inserted in the luciferase reporter gene constructs. (**E**) Interaction between miR-338 and CST3 mRNA determined by luciferase reporter gene assay. HEK293 cells were transfected with miR-338 mimic, NC mimic, the luciferase constructs of the WT CST3-3′UTR or the MUT CST3-3′UTR. After incubation for 72 h, the luciferase activity was analysed. Data are shown as mean ± SEM of three independent experiments. *P < 0.05.

Expression of CysC, the human protein encoded by CST3 gene, was detected in human primary esophageal cancer tissues and matched adjacent normal tissues by Western blotting. The results showed that CystC was robustly upregulated in the cancerous tissue compared with the adjacent (Figure 1A). To explore whether CST3 could be regulated by miRNAs, we first analysed the 3′-untranslated region (3′-UTR) of CST3 using the online server TargetScan. The output depicted that miR-338 could potentially target CST3 mRNA 3′-UTR in human (Figure 1B). To investigate whether CST3 is a target gene of miR-338, we first detected the expression levels of miR-338 in 30 primary esophageal cancer patients. The real-time qPCR analysis displayed that miR-338 was sharply decreased in the cancerous tissue compared with the adjacent (Figure 1C). Similarly, the data from 26 patients with advanced esophageal carcinoma also indated that miR-338 was dramatically decreased in the cancerous tissue (Supplementary Figure 1). Then, a wild type (WT) and a mutated (MUT, at the putative binding site in CST3 mRNA sequence, Figure 1D) CST3 3′-UTR luciferase reporter gene vectors were constructed and 3′-UTR luciferase reporter gene assay was applied to verify the interact between miR-338 and CST3 mRNA. The results showed that the miR-338 mimic transfection sharply reduced the luciferase activity of the WT vector but could not changed that of the MUT vector (Figure 1E).

**Figure 2:**
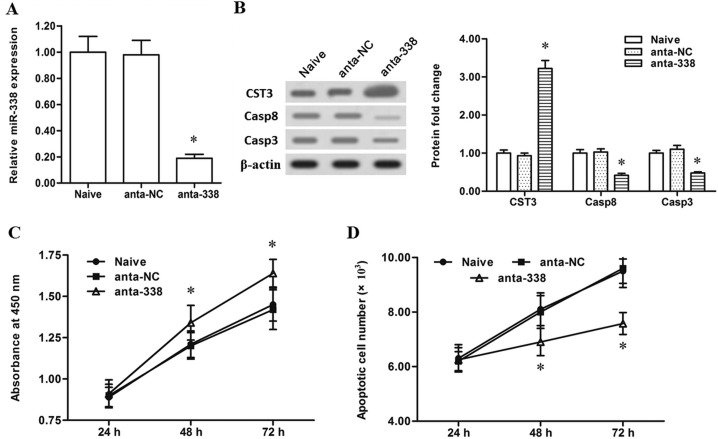
Slience of miR-338 promoted the proliferation and decreased the apoptosis of esophageal cells (**A**) MiR-338 expression was sharply reduced by the miR-338 antagomir transfection. (**B**) Slience of miR-338 greatly increased the CST3 protein expression and suppressed caspase-8/3 expression. (**C**) Slience of miR-338 promoted the proliferation of esophageal cancer cells. (**D**) Slience of miR-338 decreased the apoptosis of esophageal cancer cells. Primary esophageal cancer cells were isolated from patients. 60 nM negative antagomir (anta-NC) or 60 nM miR-338 antagomir (anta-338) was respectively transfected into the cells. After incubation for 72 h, miR-338 expression level was detected with real-time qPCR, and expression levels of CST3, caspase-8 and caspase-3 proteins were detected with Western blotting. At 24 h, 48 h and 72 h, cell proliferation and apoptosis were respectively checked with CCK-8 and FASC assays. Data are shown as mean ± SEM of four independent experiments. *P < 0.05.

**Figure 3:**
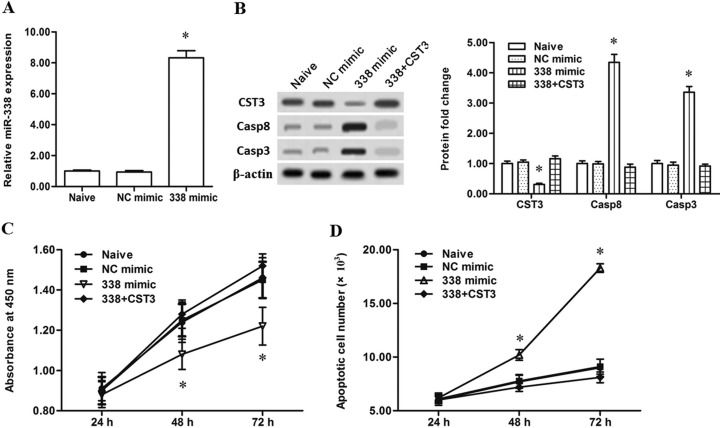
Overexpression of miR-338 attenuated the proliferation and increased the apoptosis of esophageal cancer cells (**A**) MiR-338 expression was robustly increased by the miR-338 mimic transfection. (**B**) Overexpression of miR-338 sharply reduced the CST3 protein expression and increased caspase-8/3 expression. (**C**) Overexpression of miR-338 suppressed the proliferation of esophageal cancer cells. (**D**) Overexpression of miR-338 reduced the apoptosis of esophageal cancer cells. Primary esophageal cancer cells were isolated from patients. 60 nM negative mimic (NC mimic), 60 nM miR-338 mimic (338 mimic), or 60 nM 338 mimic combined with 4 μg of pcDNA-CST3 overexpression vector was respectively transfected into the primary esophageal cancer cells. After incubation for 72 h, miR-338 expression level was detected with real-time qPCR, and expression levels of CST3, caspase-8 and caspase-3 proteins were detected with Western blotting. At 24 h, 48 h and 72 h, cell proliferation and apoptosis were respectively checked with CCK-8 and FASC assays. Data are shown as mean ± SEM of four independent experiments. *P < 0.05.

Then, to further validate the regulation of miR-338 on CST3 expression, the miR-338 antagomir and miR-338 mimic were respectively transfected into the human primary esophageal cancer cells. Then, expression levels of miR-338 and CST3, as well as the downstream pro-apoptotic genes caspase-8 and caspase-3, were detected. The results showed that the miR-338 antagomir transfection decreased the miR-338 level by ~80% (Figure 2A) and caused a ~2-fold increase in the level of CystC (Figure 2B). And, as a result, caspase-8 and caspase-3 levels were both decreased by more than a half (Figure 2B). These results indicated that CST3 is a target gene of miR-338. Additionally, CCK-8 cell proliferation assay and Annexin V-FITC/PI apoptosis detection showed that silencing miR-338 significantly increased proliferation and decreased apoptosis of the primary esophageal cancer cells (Figure 2C and 2D). In contrast to the results from the miR-338 antagomir transfection, the miR-338 mimic transfection robustly elevated the level of miR-338 and sharply reduced CystC expression (Figure 3A and 3B). Additionally, miR-338 overexpression significantly reduced proliferation and enhanced apoptosis of the primary esophageal cancer cells (Figure 3C and 3D). These data indicated that miR-338 could increase the apoptosis of esophageal cancer cells and played a suppressive role in esophageal cancer cell growth.

### LncRNA-Snhg1 played a positive role in the expression of CST3 and growth of esophageal cancer cell

**Figure 4:**
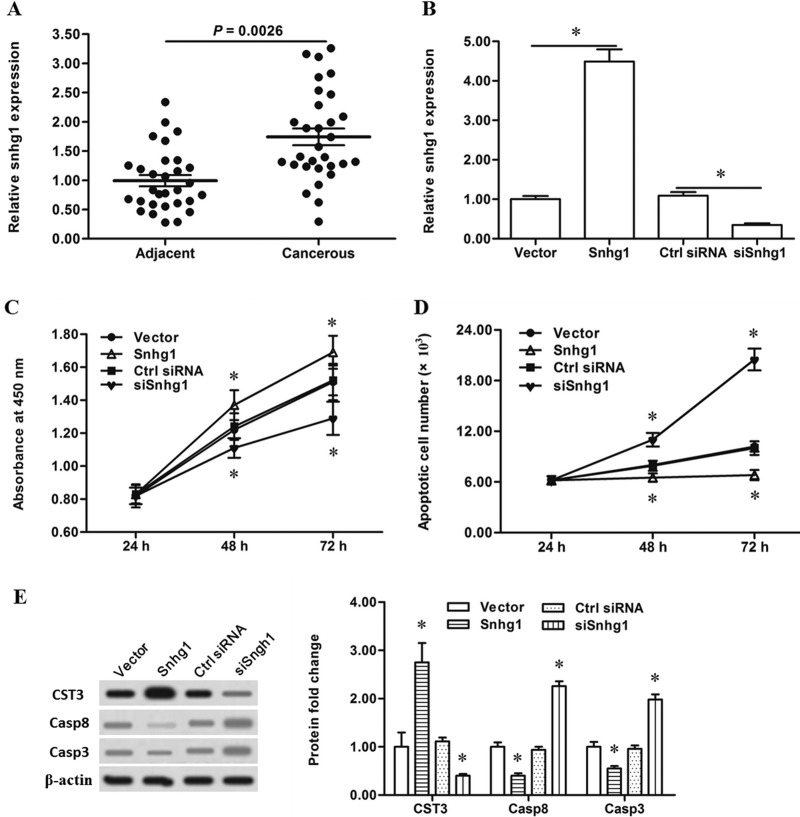
LncRNA-Snhg1 promoted esophageal cancer cell proliferation and CST3 expression (**A**) LncRNA-Snhg1 was upregulated in the esophageal carcinoma tissue. Esophageal carcinoma tissues and matched adjacent tissues were isolated from 30 patients with primary esophageal squamous cell carcinoma. LncRNA-Snhg1 expression was detected with qPCR. Then, 4 μg of pcDNA3.1 empty vector, 4 μg of pcDNA-Snhg1 overexpression vector, 60 nM control siRNA, or 60 nM Snhg1 siRNA was respectively transfected into primary esophageal cancer cells. After incubation for 72 h, lncRNA-Snhg1 expression level was detected with real-time qPCR, and expression levels of CST3, caspase-8 and caspase-3 proteins were detected with Western blotting. At 24 h, 48 h and 72 h, cell proliferation and apoptosis were respectively checked with CCK-8 and FASC assays. (**B**) LncRNA-Snhg1 expression was greatly increased by the pcDNA-Snhg1 transfection and sharply reduced by the Snhg1 siRNA transfection. **(C**) LncRNA-Snhg1 positively regulated the proliferation of esophageal cancer cells. (**D**) LncRNA-Snhg1 negatively regulated the apoptosis of esophageal cancer cells. (**E**) LncRNA-Snhg1 positively regulated the CST3 protein expression and negatively regulated caspase-8/3 expression. Data are shown as mean ± SEM of four independent experiments. *P < 0.05.

To investigate the underlying mechanism responsible for miR-338 downregulation in esophageal cancer, we wondered whether any lncRNAs, which had been shown to act as competing endogenous RNA (ceRNA) to sponge miRNAs, were involved in the regulation of miR-338 expression and miR-338 related esophageal cancer cell apoptosis. We screened some lncRNAs in the lung from a result of lncRNA microarray performed by Agilent Technologies (Santa Clara, CA). Among these lncRNAs, lncRNA-Snhg1 was significantly upregulated in cancerous tissue compared with matched adjacent both in the 30 primary esophageal cancer patients (Figure 4A) and the 26 patients with advanced esophageal carcinoma (Supplementary Figure 2). Then Snhg1 was overexpressed by the pcDNA-Snhg1 or silenced by the Snhg1 siRNA transfection in the primary esophageal cancer cells (Figure 4B). Cell proliferation and apoptosis assayes showed that Snhg1 overexpression significantly increased proliferation and decreased apoptosis of the cells, while silencing Snhg1 reduced proliferation and markedly exacerbated apoptosis of the cells (Figure 4C and 4D). In consistent with the results of cell proliferation and apoptosis assayes, Snhg1 overexpression increased the levels of CystC and diminished the levels of caspase-8/3, while silencing Snhg1 decreased the levels of CystC and elevated the levels of caspase-8/3 (Figure 4E). These data proved that lncRNA-Snhg1 played a positive role in the expression of CST3 and growth of esophageal cancer cells, which was opposite to that of miR-338.

### LncRNA-Snhg1 directly bound with miR-338 but funtioned as a non-degradable sponge

**Figure 5:**
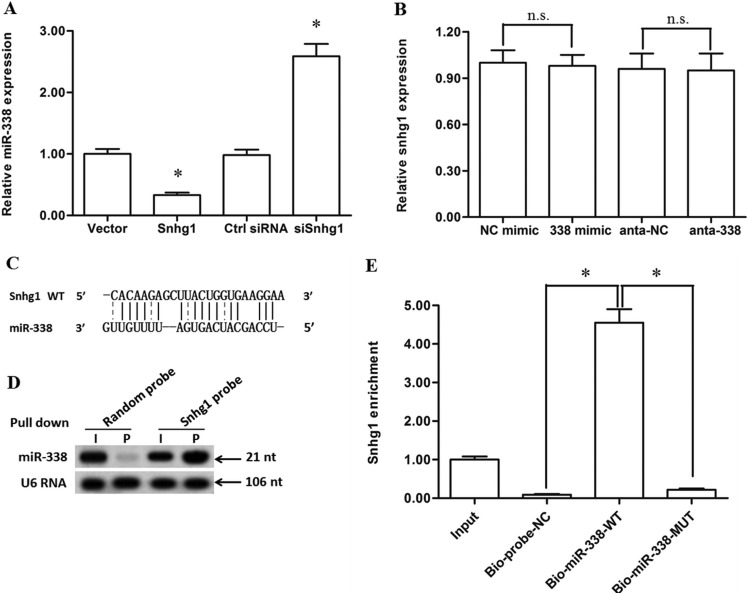
LncRNA-Snhg1 directly bound with miR-338 and acted as a non-degradable sponge for miR-338 4 μg of pcDNA3.1 empty vector, 4 μg of pcDNA-Snhg1 overexpression vector, 60 nM control siRNA, or 60 nM Snhg1 siRNA was respectively transfected into primary esophageal cancer cells. After incubation for 72 h, expression levels lncRNA-Snhg1 and miR-338 were detected with real-time qPCR. (**A**) LncRNA-Snhg1 overexpression decreased miR-338 expression, and silence of lncRNA-Snhg1 elevated miR-338 expression. (**B**) Overexpression or silence of miR-338 neither altered the lncRNA-Snhg1 level. (**C**) RNAhybrid was used to calculate the potential binding capacity of lncRNA-Snhg1 and miR-338. (**D**) LncRNA-Snhg1 could bind directly to miR-338 *in vivo*. Primary esophageal cancer cells were transfected with biotinylated Snhg1 probe or random probe-coated magnetic bead. Forty-eight hours after transfection, cells were harvested for biotin-based pull-down assay. MiR-338 expression was detected with Northern blotting. I, input (10% samples were loaded); P, pellet (100% samples were loaded). (**E**) MiR-338 could bind directly to lncRNA-Snhg1 *in vivo*. Primary esophageal cancer cells were transfected with biotinylated miR-338 or biotinylated MUT miR-338. A biotinylated miRNA that is not complementary to Snhg1 was used as a negative control (Bio-probe-NC). Forty-eight hours after transfection, cells were harvested for biotin-based pull-down assay. LncRNA-Snhg1 expression levels were analysed by real-time qPCR. Data are shown as mean ± SEM of four independent experiments. *P < 0.05.

**Figure 6:**
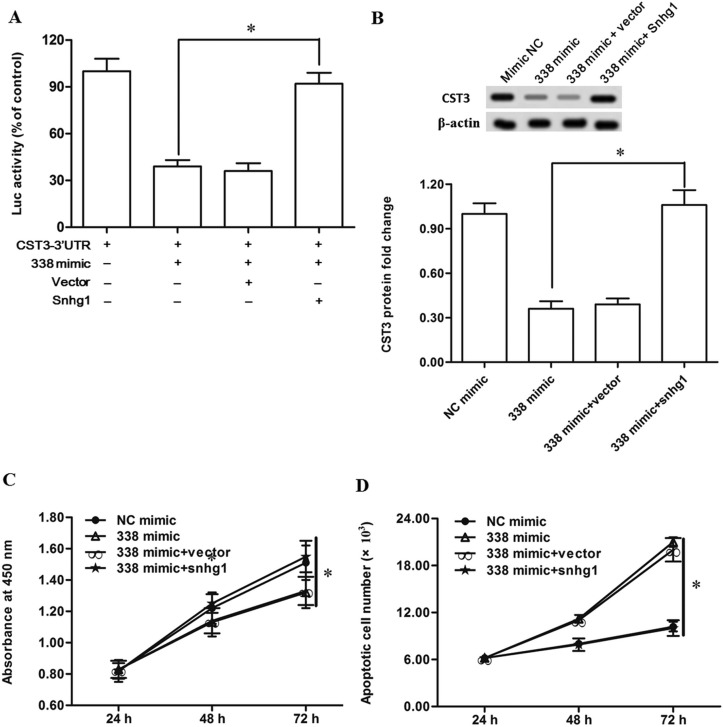
LncRNA-Snhg1 rescued the suppression of esophageal cancer cell growth and CST3 expression caused by miR-338 (**A**) LncRNA-Snhg1 inhibited the activity miR-338. Primary esophageal cancer cells were transfected with pcDNA- Snhg1 or empty vector, then transfected with miR-338 and CST3-3′UTR. Luciferase activity was analysed. (**B**) LncRNA-Snhg1 rescued the suppression of CST3 expression caused by miR-338. (**C**) LncRNA-Snhg1 rescued the suppression of esophageal cancer cell proliferation caused by miR-338. (**D**) LncRNA-Snhg1 inhibited the increase of cell apoptosis caused by miR-338. Primary esophageal cancer cells were transfected with pcDNA-Snhg1 or empty vector, then transfected with miR-338 mimic. Expression of CST3 protein was detected with Western blotting. Cell proliferation and apoptosis were respectively checked with CCK-8 and FASC assays. Data are shown as mean ± SEM of four independent experiments. *P < 0.05.

We detected the level change of miR-338 after Snhg1 overexpression and silence, as well as the level change of Snhg1 after miR-338 overexpression and silence. The results showed that Snhg1 overexpression caused a sharp decrease in the miR-338 level, while Snhg1 silence markedly increased the miR-338 level (Figure 5A). It was very interesting that, however, miR-338 overexpression or silence did not change the level of Snhg1 (Figure 5B). To investigate whether lncRNA-Snhg1 could interact with miR-338, we compared the sequence of lncRNA-Snhg1 with that of miR-338 by using the bioinformatics programme RNAhybrid. We noticed that lncRNA-Snhg1 potentially bound with miR-338 but in an incompletely matched manner (Figure 5C). Then, pull-down assays with Snhg1 biotinylated cDNA probe and biotinylated miR-338 probe were respectively performed to verify their direct binding. Compared with the random probe, miR-338 was pulled down and detected in the Snhg1 biotinylated cDNA probed RNA-RNA complexes by Western blotting (Figure 5D). Besides, the WT biotinylated miR-338 probe could pull down Snhg1, but the MUT biotinylated miR-338 probe could not (Figure 5E). Moreover, the CST3 3′-UTR luciferase reporter gene assay indicated that Snhg1 overexpression could rescued the luciferase activity suppressed by the miR-338 mimic (Figure 6A). These data indicated that lncRNA-Snhg1 could directly sponge miR-338 but it could be degraded by miR-338.

### LncRNA-Snhg1 could rescue the suppression of esophageal cancer cell growth and CST3 expression caused by miR-338

To validate the sponge effect of Snhg1 on miR-338 induced suppression of CST3 expression and esophageal cancer cell growth, the miR-338 mimic was transfected alone or co-transfected with the pcDNA-Snhg1 into primary esophageal cancer cells. Western blotting analysis showed that Snhg1 overexpression rescued the suppression of CST3 expression (Figure 6B). Moreover, Snhg1 overexpression also rescued the growth suppression and apoptosis promotion caused by the miR-338 mimic transfection (Figure 6C and 6D).

## DISCUSSION

Several recent studies showed that miR-338 was downregulated in esophageal squamous cell carcinoma tissue, suggesting it a role in progression of esophageal cancer [20, 21]. In this study, we also found that miR-338 displayed a 30% decreased expression in esophageal squamous cell carcinoma tissues compared with the adjacent, and acted as a suppressor in esophageal carcinoma cell growth.

The role of miRNAs usually depends on what genes they target. Up to date, it has been shown that miR-338 respectively targeted proto-oncogenes smoothened (SMO) [17], Phosphatidylinositol-3,4,5-trisphosphate-dependent Rac exchange factor 2 (PREX2a) [22], synovial sarcoma and X breakpoint 2 interacting protein (SSX2IP) [19] respectively in several solid tumors. In this study, we predicted and identified proto-oncogene CST3 as a target gene of miR-338 in esophageal carcinoma cells. Gain-and-loss-function experiments indicated that miR-338 suppressed expression of CST3 protein, and promoted expression of apoptotic proteins caspase-8/3.

Recently, the roles of CysC in cerebrovascular disorders and carcinogenesis were revealed [23–25]. Its exact role in carcinogenesis is still inconclusive. As the strongest cathepsin B inhibitor, CysC was proven to be a pro-apoptotic and growth suppressive factor, and has been applied in the diagnosis and prognosis of some cancers [26, 27]. Besides, it was also reported that CysC could antagonizes multiple key factors involved in promotion of cell invasion/migration, such as transforming growth factor-β (TGF-β) and matrix metalloproteinases (MMPs) [28, 29]. Therefore, it is believed that CysC played a negative role in cancer invasion and migration, at least in some types of cancers. However, some other studies indicated that high serum CysC level was related with poor prognosis and it could promote the progression of several cancers [24, 30]. Moreover, cathepsin B, one of major cathepsins inhibited by CysC, was demonstrated to be a protease with caspase-processing activity and an pro-apoptotic and –inflammatory cathepsin member [6, 31]. In our previous study, we reported that CST3 mRNA and protein levels were significantly increased in esophageal carcinoma tissues compared with the normal adjacent tissues [32]. In this current study, we also found that CysC protein was significantly upregulated in esophageal carcinoma tissues. We demonstrated that CysC increased esophageal carcinoma cell growth and suppressed cell apoptosis.

Most recently, a couple of studies revealed that lncRNA-Snhg1 predicted a poor prognosis and promoted cell growth in nonsmall cell lung cancer and hepatocellular carcinoma [33, 34]. Here, in esophageal cancer, we also found that lncRNA-Snhg1 was significantly upregulated in the cancerous tissues compared with matched adjacent tissue, had a promotive effect on cell proliferation, and suppressed the cell apoptosis. Moreover, our results from bioinformatics, luciferase reporter gene and RNA pull-down assays indicated that Snhg1 could sponge miR-338 to relieve it suppression on the proto-oncogene CST3.

CeRNA is a fine regulatory mode of gene expression proposed several years before. In this mode, lncRNAs or circRNAs regulate other RNA transcripts, usually a protein coding ones, by competing for shared miRNAs. With deep understanding of this mode, researches raised the premise that the mode functioned in the cell: the ceRNA molecule and the shared miRNA should be both located in the cytoplasm [35, 36]. In this study, the ceRNA molecule lncRNA-Snhg1 and the shared miRNA miR-338 were previously demonstrated to be expressed in cytoplasm [37, 38]. In reported ceRNA modes, lncRNAs were targeted by specific miRNAs and they negatively regulated expression of each other, which means that lncRNAs usually act as degradable sponges for miRNAs. However, it was interesting that lncRNA-Snhg1 sponged miR-338 in esophageal cancer cells but it was non-degradable.

In conclusion, lncRNA-Snhg1 promotes cell proliferation by acting as a non-degradable sponge for the tumor suppressor miR-338 in esophageal cancer cells.

## MATERIALS AND METHODS

### Ethics statement and sampling

30 patients with early primary and 26 patients with advanced esophageal squamous cell carcinoma were enrolled in the study. They were aged from 35 to 55 years with an equal number of males and females. The 30 patients with early primary carcinoma had no evidence of regional lymph node metastasis and the lengths of their cancerous tissues in cervicum or thorax were less than 2.5 cm. Besides, none of the patients had received preoperative anticancer treatment. The esophageal tissue of the 26 patients with advanced carcinoma is infiltrated with surrounding tissues accompanied by a wide range of lymphatic metastasis. Cancerous tissues and matched adjacent normal esophageal epithelial tissues were sampled from endoscopic biopsies. The study was approved by the Ethical Committee of The First Affiliated Hospital of Zhengzhou University. The patients were previously informed of the experimental details and gave written consents.

### Isolation and culture of primary esophageal cancer cells

Esophageal squamous cell carcinoma cells were isolated from 6 patients(3 male, 3 female; age range 40–50 years) with early primary esophageal squamous cell carcinoma by endoscopic biopsy. Cell isolation was performed by incubation (30 min) with a solution containing 0.05% trypsin, 0.02% EDTA and 0.05% (w/v) collagenase. The isolated cells were maintained in RPMI21640 supplemented with 20% FBS, 2 mM L-glutamine, 2% Biotect protective medium, penicillin (100 U/mL), streptomycin (100 mg/mL), 1% amphotericin B. The cells were incubated at 37°C in a humidified and 5%-CO_2_atmosphere.

### Constructs, sythesized oligos and transfection

Snhg1 sequence was PCR amplified and inserted into the pcDNA3.1 (+) vector at the sites of Kpn I and BamH I (Invitrogen). The primers used were as follows: forward 5′-GGG GTA CCG TTC TCA TTT TTC TAC TGC TCG TG-3′ and reverse 5′-CGG GAT CCA TGT AAT CAA TCA TTT TAT TAT TTT CAT C-3′. The Snhg1 siRNAs, miR-338 mimics and miR-338 antagomirs, as well as scrambled negative control oligos, were synthesized by Ribobio (Guangzhou, China). The cells were transfected with constructs or oligos using Lipofectamine 3000 (Invitrogen) according to the manufacturer's instructions.

### Real-time quantitative PCR

Total RNA was extracted with the Trizol agent. After concentration, purity and integrity detections, 2 μg of DNase I-treated RNA was applied in the reverse transcription reaction with 2 mM specific RT primers and superscript III reverse transcriptase (Invitrogen). Real-time qPCR reactions were carried out in a final volume of 25 μL, using SYBR Premix Ex Taq (TaKaRa), 0.4 mM of each primer (forward and bottom), and 200 ng of obtained cDNA template. Each individual sample was run in triplicate wells. PCR amplification cycles were performed using the iQ^TM^5 Multicolor Real-Time PCR Detection System (Bio-Rad) and SYBR Premix Ex Taq II kit (TaKaRa). The reaction profile was as follows: 95°C for 3 min followed by 30 cycles of 95°C for 10 s, 50°C for 30 s, 72°C for 20 s. The change in transcript abundance of all tested genes was calculated using the 2^−ΔΔCt^ method. All gene mRNA amounts were normalized to U6 RNA. The primers used in the reactions were respectively as follows: miR-338, RT primer 5′-GTC GTA TCC AGT GCA GGG TCC GAG GTG CAC TGG ATA CGA CCA ACA AAA-3′, Forward 5′-TGC GGT CCA GCA TCA GTG AT-3′, Reverse 5′-CCA GTG CAG GGT CCG AGG T-3′; U6 RT primer 5′-GTC GTA TCC AGT GCA GGG TCC GAG GTG CAC TGG ATA CGA CAA AAT ATG G-3′, U6 Forward 5′-TGC GGG TGC TCG CTT CGG CAG C-3′, U6 Reverse 5′-CCA GTG CAG GGT CCG AGG T-3′; lncRNA-Snhg1 forward 5′-AGG CTG AAG TTA CAG GTC-3′ and Reverse 5′-TTG GCT CCC AGT GTC TTA-3′.

### Detection of cell proliferation and apoptosis

The cell proliferation assay was performed using a Cell Counting Kit-8 (Sigma) according to the manufacturer's instructions. The data were expressed as the absorbance at 450 nm.

Fluorescence-activated cell sorter (FACS) was used to investigate cell apoptosis. Post treatment, the cells were collected, washed in PBS, and then stained by using Annexin V-FITC/PI cell apoptosis detection kit (San Francisco, CA, USA) and analyzed with a flow cytometer (ACSCalibur, Becton Dickinson, San Jose, CA).

### Western blotting

Twenty micrograms of protein was separated by 12% SDS-PAGE and electro-transferred onto a 0.22 μm PVDF membrane (Millipore) for immunoblotting analysis. The following primary antibodies were used: anti-Cyst 3 (1:300, Abcam), anti-caspase 3 (acetyl K382) (1:200, Abcam), anti-caspase 8 (1:400, Abcam), and anti-β-actin (1:800, Santa Cruz) that was used as the internal reference. After incubation with the appropriate HRP-conjugate secondary antibodies, proteins were detected using an Odyssey infrared scanner (Li-Cor Biosciences).

### Luciferase constructs and activity detection

CST3 3′UTR or Snhg1 transcript were amplified by PCR. To produce mutated 3′UTR, the mutations were generated using QuikChange II XL Site-Directed Mutagenesis Kit (Stratagene). Wild type and mutated 3′UTRs were subcloned into the pGL3 vector (Promega) immediately downstream the coding region of luciferase gene. HEK293T cells were infected with the indicated oligos or/and constructs, then transfected with the indicated luciferase vectors as described in the corresponding figure legends. The transfection was performed using Lipofectamine 3000 (Invitrogen) according to the manufacturer's instructions. The luciferase activity was assayed as described elsewhere [39].

### Pull-down assay with biotinylated cDNA probe

The biotinylated DNA probe complementary to Snhg1 was synthesized and dissolved in 500 μL of wash/binding buffer (0.5 M NaCl, 20 mM Tris-HCl, pH 7.5, and 1 mM EDTA). The probes were incubated with streptavidin-coated magnetic beads (Sigma) at 25°C for 2 h to generate probe-coated magnetic beads. The esophageal cancer cell lysates were incubated with probe-coated beads. After washing with the wash/binding buffer, the RNA complexes bound to the beads were eluted and extracted for northern blot analysis. Snhg1 pull-down probe, 5′-TAA CAA CTA ACT TGA AGG GTA TT-3′, and random pull-down probe, 5′-TGA TGT CTA GCG CTT GGG CTT TG-3′.

### Northern blot analysis

The cell samples were collected and run on a 15% polyacrylamide-urea gel, transferred to positively charged nylon membranes (Millipore) followed by cross-linking through UV irradiation. The membranes were subjected to hybridization with 100 pmol 30-digoxigenin (DIG)-labelled probes overnight at 42°C. Probes were labelled with DIG using a 3′-End DIG Labelling Kit (Roche). The detection was performed using a DIG luminescent detection kit (MyLab) according to the manufacturer's instructions. The probe sequence for miR-338-3p was 5′-AGG TCG TAG TCA CTA AAA CAA C-3′. U6 (internal control) probe sequence was 5′-GCT AAT CTT CTC TGT ATC GTT CC-3′.

### Pull-down assay with biotinylated miRNA

The esophageal cancer cells were transfected with biotinylated miR-338 or the mut (50 nM), and were harvested 48 h after transfection. The cells were washed with PBS followed by brief vortex, and incubated in a lysis buffer (20 mM Tris, pH 7.5, 200 mM NaCl, 2.5 mM MgCl2, 0.05% Igepal, 60 U ml 1 Superase-In (Ambion), 1 mM DTT, protease inhibitors (Roche) on ice for 10 min. The lysates were precleared by centrifugation, and 50 μL of the sample was aliquoted for input. The remaining lysates were incubated with M-280 streptavidin magnetic beads (Sigma). To prevent non-specific binding of RNA and protein complexes, the beads were coated with RNase-free BSA and yeast tRNA (both from Sigma). The beads were incubated at 4°C for 3 h, washed twice with ice-cold lysis buffer, three times with the low salt buffer (0.1% SDS, 1% Trition X-100, 2 mM EDTA, 20 mM TrisHCl pH8.0 and 150 mM NaCl) and once with the high salt buffer (0.1% SDS, 1% Trition X-100, 2 mM EDTA, 20 mM Tris-HCl pH 8.0 and 500 mM NaCl). The bound RNAs were purified by Trizol and then applied in the qPCR analysis for lncRNA-Snhg1 enrichment.

### Statistical analysis

All data were obtained from at least three independent experiments. Values were expressed as means ± SEM. Statistics were calculated with SPSS statistics v19.0 software. Multiple comparisons were assessed by one-way ANOVA followed by Dunnett's tests. The difference between groups was considered statistically significant if *P* < 0.05.

## SUPPLEMENTARY MATERIALS FIGURES


