# Supplementary material for: LncRNA Snhg1, a non-degradable sponge for miR-338, promotes expression of proto-oncogene CST3 in primary esophageal cancer cells
Source: Oncotarget. 2017 Mar 14;8(22):35750–60. doi: 10.18632/oncotarget.16189 (PMC5482614; doi:10.18632/oncotarget.16189)
Supplement: Supplementary file 1 [file oncotarget-08-35750-s001.pdf]

LncRNA Snhg1, a non-degradable sponge for miR-338, promotes expression of proto-oncogene CST3 in primary esophageal cancer cells

Supplementary Materials

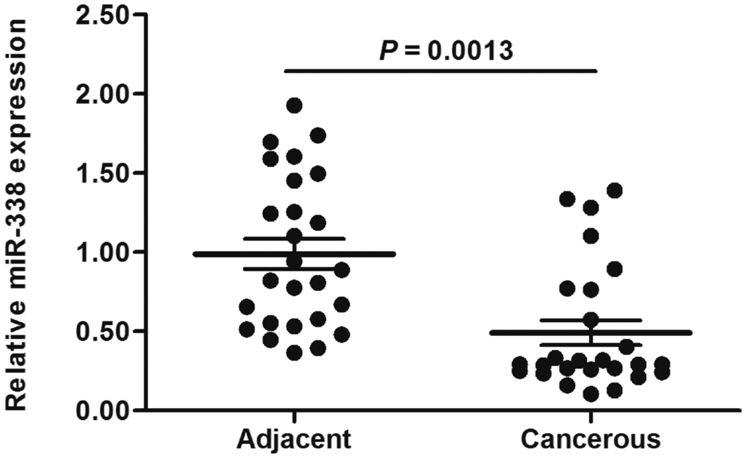

Supplementary Figure 1: MiR-338 was downregulated in the esophageal carcinoma tissue. Esophageal carcinoma tissues and matched adjacent tissues were isolated from 26 patients with advanced esophageal squamous cell carcinoma. MiR-338 expression was detected with qPCR.

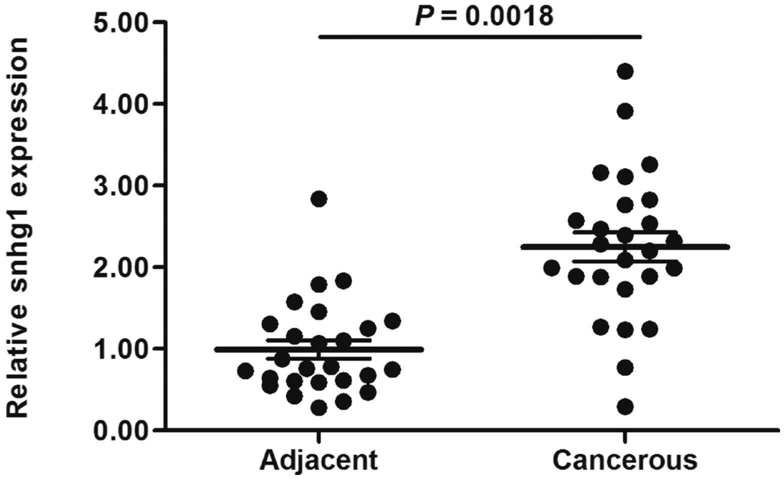

Supplementary Figure 2: LncRNA-Snhg1 was upregulated in the esophageal carcinoma tissue. Esophageal carcinoma tissues and matched adjacent tissues were isolated from 26 patients with advanced esophageal squamous cell carcinoma. LncRNA-Snhg1 expression was detected with qPCR.
